# Inhibition of Fatty Acid Binding Proteins Elevates Brain Anandamide Levels and Produces Analgesia

**DOI:** 10.1371/journal.pone.0094200

**Published:** 2014-04-04

**Authors:** Martin Kaczocha, Mario J. Rebecchi, Brian P. Ralph, Yu-Han Gary Teng, William T. Berger, William Galbavy, Matthew W. Elmes, Sherrye T. Glaser, Liqun Wang, Robert C. Rizzo, Dale G. Deutsch, Iwao Ojima

**Affiliations:** 1 Department of Anesthesiology, Stony Brook University, Stony Brook, New York, United States of America; 2 Department of Biochemistry and Cell Biology, Stony Brook University, Stony Brook, New York, United States of America; 3 Department of Chemistry and the Institute of Chemical Biology and Drug Discovery, Stony Brook University, Stony Brook, New York, United States of America; 4 Department of Biological Sciences, Kingsborough Community College, Brooklyn, New York, United States of America; 5 Department of Applied Mathematics and Statistics, Stony Brook University, Stony Brook, New York, United States of America; University of Arizona, United States of America

## Abstract

The endocannabinoid anandamide (AEA) is an antinociceptive lipid that is inactivated through cellular uptake and subsequent catabolism by fatty acid amide hydrolase (FAAH). Fatty acid binding proteins (FABPs) are intracellular carriers that deliver AEA and related N-acylethanolamines (NAEs) to FAAH for hydrolysis. The mammalian brain expresses three FABP subtypes: FABP3, FABP5, and FABP7. Recent work from our group has revealed that pharmacological inhibition of FABPs reduces inflammatory pain in mice. The goal of the current work was to explore the effects of FABP inhibition upon nociception in diverse models of pain. We developed inhibitors with differential affinities for FABPs to elucidate the subtype(s) that contributes to the antinociceptive effects of FABP inhibitors.

Inhibition of FABPs reduced nociception associated with inflammatory, visceral, and neuropathic pain. The antinociceptive effects of FABP inhibitors mirrored their affinities for FABP5, while binding to FABP3 and FABP7 was not a predictor of *in vivo* efficacy. The antinociceptive effects of FABP inhibitors were mediated by cannabinoid receptor 1 (CB_1_) and peroxisome proliferator-activated receptor alpha (PPARα) and FABP inhibition elevated brain levels of AEA, providing the first direct evidence that FABPs regulate brain endocannabinoid tone. These results highlight FABPs as novel targets for the development of analgesic and anti-inflammatory therapeutics.

## Introduction

Fatty acid binding proteins (FABPs) comprise a family of small cytoplasmic lipid transport proteins [1]. FABPs are expressed in numerous tissues, including the central and peripheral nervous systems [2]–[6] and bind to a subset of endogenous ligands including fatty acids, retinoic acid, and N-acylethanolamines (NAEs) [7]–[10]. The endocannabinoid anandamide (AEA) is an NAE that activates cannabinoid receptors (CB) while palmitoylethanolamide (PEA) and oleoylethanolamide (OEA) signal through nuclear peroxisome proliferator-activated receptor alpha (PPARα) [11]–[13].

FABPs regulate a plethora of physiological processes including lipid metabolism, neurite outgrowth, inflammation, sleep, and neuronal signaling [14]–[20]. Consequently, modulation of FABP function may hold therapeutic promise for the treatment of diverse disorders. Indeed, genetic or pharmacological inhibition of FABPs protects against atherosclerosis, diet induced obesity, experimental autoimmune encephalomyelitis and ameliorates dyslipidemias [20]–[22]. These effects are mediated through distinct targets including kinases, PPAR gamma, and through attenuation of pro-inflammatory cytokine release [20], [23]–[25].

We have previously demonstrated that FABP5 and FABP7 are capable of binding to NAEs including AEA and OEA and regulate their signaling and catabolism by fatty acid amide hydrolase (FAAH), the principal NAE hydrolyzing enzyme in mice [8], [9], [26]. Previous work has established that inhibition of FAAH potentiates NAE signaling at CB_1_, CB_2_, and PPARα receptors and produces antinociceptive and anti-inflammatory effects in models of visceral, inflammatory, and neuropathic pain [26]–[29]. Similar effects are observed following inhibition of monoacylglycerol lipase, the major enzyme that hydrolyzes the endocannabinoid 2-arachidonoylglycerol (2-AG) [30]. These data indicate that targeting endocannabinoids and NAEs may offer a therapeutic avenue for the treatment of pain and inflammation.

Recently, we developed a novel α-truxillic acid-based FABP inhibitor termed SBFI26 and demonstrated that pharmacological FABP inhibition reduced nociception and inflammation in the formalin and carrageenan models of pain [31]. Here, we evaluate three new analogs based on SBFI26 to determine how inhibition across FABP3, FABP5, and FABP7 would reduce nociception associated with models of visceral, inflammatory, and neuropathic pain. Furthermore, we examined the role of CB and PPARα receptors in these processes and determined whether FABP inhibition elevates NAE and endocannabinoid levels in mouse brain.

## Materials and Methods

### Ethics Statement

The experiments conducted herein conform to the National Institutes of Health Guidelines for the Care and Use of Laboratory Animals and were approved by the Stony Brook University Institutional Animal Care and Use Committee (IACUC #2011-1834).

### Chemicals

12-NBD-stearate [12-*N*-methyl-(7-nitrobenz-2-oxa-1,3-diazo)aminostearicacid] was from Avanti Polar Lipids (Alabaster, AL). PEA, *d_4_*-PEA, OEA, *d_2_*-OEA, 2-AG and *d_5_*-2-AG were from Cayman Chemical (Ann Arbor, MI). AEA and *d_4_*-AEA were from R&D systems. [^14^C]AEA (arachidonoyl-[1-^14^C]ethanolamide, 60 mCi/mmol) was provided by the Drug Supply Program at the National Institute on Drug Abuse while [^3^H]2-oleoylglycerol ([^3^H]2-OG) was purchased from Perkin Elmer. Acetonitrile, chloroform, and methanol were of the highest possible purity and were obtained from Fisher Scientific.

### Synthesis of SBFI26, SBFI50, SBFI60, and SBFI62

#### Synthesis of SBFI26 (alpha-2,4-diphenylcyclobutane-1,3-dicarboxylic acid mono-1-naphthyl ester)

Alpha-truxillic acid (297 mg, 1.0 mmol) was suspended in thionyl chloride (3 mL), and one drop of DMF was added to the suspension. The reaction mixture was heated to reflux for 3 h. The excess thionyl chloride and DMF was removed *in vacuo* and truxillic acyl chloride was obtained, which was used directly in the subsequent reaction. To the solution of truxillic acyl chloride in THF (10 mL) was added drop wise the solution of 1-naphthol (120 mg, 0.84 mmol) in THF (5 mL) and pyridine (0.5 mL), and the reaction mixture was heated to reflux for 3 h. The reaction was quenched with addition of water (2 mL). The resulted solution was diluted with ethyl acetate (15 mL) and the aqueous layer was separated. The organic layer was dried over MgSO_4_ and concentrated *in vacuo*. The crude product was purified by flashed column chromatography on silica gel using ethyl acetate/hexanes (10% → 25%) as eluent to afford alpha-truxillic acid mono-1-naphthyl ester as white solid (202 mg, 57%): m.p.: 192–193 °C; ^1^H NMR (300 MHz, (CD_3_)_2_CO) δ 4.15 (dd, *J* = 10.4, 7.0 Hz, 1H), 4.56 (dd, *J* = 10.4, 7.0 Hz, 1H), 4.75–4.68 (m, 2H), 6.42 (d, *J* = 7.5 Hz, 1H), 7.65–7.24 (m, 15H), 7.72 (d, *J* = 8.2 Hz, 1H); ^13^C NMR (300 MHz, (CD_3_)_2_CO) δ 41.650, 42.119, 45.416, 46.766, 117.932, 121.361, 125.251, 125.678, 126.270, 126.724, 126.849, 126.921, 127.396, 127.629, 127.826, 128.245, 128.271, 128.490, 128.754, 129.055, 134.473, 139.456, 170.680, 172.139; HRMS (ES) m/z calculated for C_28_H_22_O_4_ + NH_4_ (M + NH_4_)^+^: 440.1862, found 440.1856 (Δ -1.2 ppm).

#### Synthesis of SBFI50 (alpha-2,4-diphenylcyclobutane-1,3-dicarboxylic acid mono-2-naphthyl ester)

Alpha-truxillic acid (297 mg, 1.0 mmol) was suspended in thionyl chloride (3 mL), and one drop of DMF was added to the suspension. The reaction mixture was heated to reflux for 3 h. The excess thionyl chloride and DMF was removed *in vacuo* and truxillic acyl chloride was obtained, which was used directly in the subsequent reaction. To the solution of truxillic acyl chloride in THF (10 mL) was added drop wise the solution of 2-naphthol (115 mg, 0.80 mmol) in THF (5 mL) and pyridine (0.5 mL), and the reaction mixture was heated to reflux for 3 h. The reaction was quenched with addition of water (2 mL). The resulted solution was diluted with ethyl acetate (15 mL) and the aqueous layer was separated. The organic layer was dried over MgSO_4_ and concentrated *in vacuo*. The crude product was purified by flashed column chromatography on silica gel using ethyl acetate/hexanes (10% → 25%) as eluent to afford alpha-truxillic acid mono-2-naphthyl ester as white solid (175 mg, 52%): m.p.: 204–206 °C; ^1^H NMR (300 MHz, (CD_3_)_2_CO) δ 4.15 (dd, *J* = 10.6, 7.2 Hz, 1H), 4.34 (dd, *J* = 10.6, 7.2 Hz, 1H), 4.71–4.58 (m, 2H), 6.58 (d, *J* = 8.8 Hz, 1H), 6.84 (s, 1H), 7.61–7.26 (m, 12H), 7.78–7.70 (m, 2H), 7.86 (d, *J* = 8.0 Hz, 1H); ^13^C NMR (300 MHz, (CD_3_)_2_CO) δ 41.295, 42.022, 46.043, 46.768, 118.430, 121.305, 125.596, 126.457, 126.909, 127.250, 127.294, 127.635, 127.808, 128.184, 128.264, 128,606, 128.870, 131.389, 133.634, 139.244, 139.349, 148.374, 170.656, 172.154; HRMS (ES) m/z calculated for C_28_H_22_O_4_ + NH_4_ (M + NH_4_)^+^: 440.1864, found 440.1856 (Δ -1.8 ppm).

#### Synthesis of SBFI60 (alpha-2,4-diphenylcyclobutane-1,3-dicarboxylic acid mono-1-naphthyl amide)

Alpha-truxillic acid (150 mg, 0.51 mmol) was suspended in thionyl chloride (3 mL), and one drop of DMF was added to the suspension. The reaction mixture was heated to reflux for 3 h. The excess thionyl chloride and DMF was removed *in vacuo* and truxillic acyl chloride was obtained, which was used directly in the subsequent reaction. To the solution of truxillic acyl chloride in THF (10 mL) was added dropwise the solution of naphthyl-1-amine (36 mg, 0.25 mmol) in THF (5 mL) and pyridine (0.2 mL), and the reaction mixture was heated to reflux for 3 h. The reaction was quenched with addition of 1 M HCl solution (2 mL). The resulted solution was diluted with ethyl acetate (15 mL) and the aqueous layer was separated. The organic layer was dried over MgSO_4_ and concentrated *in vacuo*. The crude product was crystallized from ethyl acetate to afford alpha-truxillic acid mono-1-naphthyl amide as white solid (72 mg, 68%): m.p.: 198–200 °C; ^1^H NMR (300 MHz, (CD_3_)_2_SO) δ 3.95 (dd, *J* = 10.4, 6.6 Hz, 1H), 4.20 (dd, *J* = 10.4, 6.6 Hz, 1H), 4.44 (m, 2H), 7.07 (d, *J* = 7.4 Hz, 1H), 7.24–7.47 (m, 14H), 7.65 (d, *J* = 8.1 Hz, 1H), 7.83 (d, *J* = 8.1 Hz, 1H), 9.81 (s, 1H), 12.11 (s, 1H); ^13^C NMR (300 MHz, (CD_3_)_2_SO) δ 40.834, 42.202, 46.384, 47.840, 122.251, 123.442, 125.652, 125.756, 125.972, 126.280, 127.102, 127.166, 128.093, 128.211, 128.374, 128.435, 128.606, 128.701, 133.841, 133.950, 139.780, 140.511, 170.419, 173.592; HRMS (ES) m/z calculated for C_28_H_23_NO_3_ + NH_4_ (M + NH_4_)^+^: 440.1862, found 440.1856 (Δ -1.2 ppm).

#### Synthesis of SBFI62 (2,4-diphenylcyclobutane-1,3-dicarboxylic acid di-1-naphthyl amide)

Alpha-truxillic acid (50 mg, 0.17 mmol) was suspended in thionyl chloride (1.5 mL), and one drop of DMF was added to the suspension. The reaction mixture was heated to reflux for 3 h. The excess thionyl chloride and DMF was removed *in vacuo* and truxillic acyl chloride was obtained, which was used directly in the subsequent reaction. To the solution of truxillic acyl chloride in THF (10 mL) was added dropwise the solution of naphthyl-1-amine (48 mg, 0.34 mmol) in THF (3 mL) and pyridine (0.2 mL), and the reaction mixture was heated to reflux for 3 h. The reaction was quenched with addition of 1 M HCl solution (2 mL). The resulted solution was diluted with ethyl acetate (15 mL) and the aqueous layer was separated. The organic layer was dried over MgSO_4_ and concentrated *in vacuo*. The crude product was crystallized from ethyl acetate to afford alpha-truxillic acid di-1-naphthyl amide as white solid (75 mg, 82%): m.p.: >240 °C; ^1^H NMR (300 MHz, (CD_3_)_2_SO) δ 4.34 (t, *J* = 7.74 Hz, 2H), 4.65 (t, *J* = 7.74 Hz, 2H), 7.09 (d, *J* = 7.4 Hz, 2H), 7.25–7.55 (m, 18H), 7.67 (d, *J* = 8.1 Hz, 2H), 7.83 (d, *J* = 8.1 Hz, 2H) 9.87 (s, 2H); ^13^C NMR (300 MHz, (CD_3_)_2_SO) δ 41.358, 47.665, 122.340, 123.526, 125.656, 125.783, 125.972, 126.286, 127.155, 128.205, 128.473, 128.624, 128.674, 133.972, 140.425, 170.654; HRMS (ES) m/z calculated for C_38_H_30_N_2_O_2_ + H (M+H)^+^:547.2386, found 547.2386 (Δ 0 ppm).

### Drugs

SBFI26, SBFI50, SBFI60, and SBFI62 were synthesized as described above. Rimonabant, SR144528, PF-3845, and naloxone were kindly provided by the Drug Supply Program at the National Institute on Drug Abuse. GW6471 was from Sigma. All drugs were injected in a volume of 10 μl/g body weight.

### Energy Scoring of SB-FI compounds with FABPs

Molecular mechanics-based interaction energy scores were computed using DOCK version 6.6. An energy grid for FABP7 (PDB: 1FE3) was previously generated with 6–9 Lennard-Jones exponents and at 0.3 Å resolution and re-docking of SBFI26 was performed [31] using the standard DOCK flexible ligand (FLX) protocol [32]. Additional target FABPs – FABP5 (PDB: 1B56), and FABP3 (PDB: 3RSW) – were aligned to FABP7 in UCSF Chimera [33] using protein backbone alpha-carbons resulting in RMSDs of 0.95 Å for FABP5 and 0.96 Å for FABP3, suggesting an overall good alignment. Structures and mol2 files of the α-truxillic acid analogs SBFI50, SBFI60, and SBFI62 were created from SBFI26 in UCSF Chimera preserving the binding pose from initial docking to the FABP7 grid. All four SB-FI ligands were then energy-minimized in each FABP pocket using parameters consistent with the DOCK rigid ligand (RGD) protocol [32]. Energy minimizations were performed in Cartesian space and with 6–12 Lennard-Jones exponents [34]. All calculations were performed on a Dell PowerEdge T110 server running Ubuntu version 12.04 and using an Intel quad-core CPU in the Rizzo laboratory (Dept. of Applied Mathematics and Statistics, Stony Brook University).

### Binding of Inhibitors to FABPs

Inhibitory constant (K_i_) values were elucidated utilizing NBD-stearate fluorescence displacement as described previously [9], [31]. The tests were performed in 96-well plates containing inhibitors (0.1 – 50.0 μM), FABP, and NBD-stearate. Fluorescence intensity was measured with a FLUOstar OPTIMA spectrofluorometer with excitation and emission wavelengths of 460 and 544 nm, respectively. K_i_ values were derived from nonlinear regression analysis using the equation K_i_  =  IC_50_/(1+([NBD-stearate]/K_d_)). Respective K_d_ values of 0.18, 0.16, and 0.22 μM for NBD-stearate with FABP3, FABP5, and FABP7 were determined previously [9].

### Enzyme Assays

AEA and 2-OG hydrolysis was determined using modified protocols to those described previously [8], [35]. Mice were injected with inhibitors (20 mg/kg, i.p.) and the brains and livers were harvested 60 min later. The tissue homogenates were subsequently incubated with 30 μM [^14^C]AEA or 10 μM [^3^H]2-OG for 30 min. Reactions were stopped with two volumes of chloroform:methanol (1∶1) and the methanol phase was counted using a Beckman LS6500 scintillation counter.

### Cytotoxicity

Cytotoxicity of the FABP inhibitors SBFI26 and BMS309403 were examined using the MTT [3-(4,5-dimethylthiazol-2-yl)-2,5 diphenyl tetrazolium bromide] colorimetric assay. HeLa cells were seeded into 96-well plates (15,000 cells/well) and incubated for 24 hours at 37°C in DMEM supplemented with 10% FBS, penicillin (100 U/mL) and streptomycin (100 μg/mL). The cells were subsequently washed with PBS and treated with serum-free DMEM containing 10 nM to 1 mM SBFI26, BMS309403, or 5 mM hydrogen peroxide as a positive control. Following a 24 hr incubation, the cells were washed with PBS and treated with MTT (0.5 mg/mL in PBS) for 4 hrs. The cells were subsequently solubilized using DMSO and the absorbance was read at 562 nm in a microplate reader.

### Animals

Male C57Bl/6 mice (22–30 g, Taconic Farms) or male Fischer 344 rats (250–350 g, Taconic Farms) were used for all experiments. The animals were group housed at room temperature and kept on a 12∶12 hour light:dark cycle with *ad libitum* access to water and food. The animals were habituated to the experimental room for one day before testing and at least two hours before each experiment. All experiments were approved by the Stony Brook University Institutional Animal Care and Use Committee. Unless otherwise stated, animals were euthanized with CO_2_ and subsequently decapitated. The experimenter was blinded to the treatment condition of each animal.

### Carrageenan-induced Paw Edema and Thermal Hyperalgesia

Paw edema was induced by injecting 1% λ-carrageenan (20 μl, in sterile saline) into the plantar surface of the left hind paw using a 30 gauge needle attached to a glass syringe (Hamilton Company). Paw diameters were measured before carrageenan injection and 4 or 24 hours after injection using digital electronic calipers (Fisher) and expressed to the nearest ± 0.01 mm. SBFI26, SBFI50, SBFI60, and SBFI62 were dissolved in DMSO:cremophor-EL:saline (4% DMSO:10% Cremophor-EL) and administered (20 mg/kg, i.p.) 60 min prior to injection of carrageenan. Receptor antagonists were injected 10 min before the inhibitors. Rimonabant and SR144528 (3 mg/kg, i.p.) were dissolved in ethanol:cremophor-EL:saline (1∶1∶18) while GW6471 (4 mg/kg, i.p.) was dissolved in DMSO:cremophor-EL:saline (2% DMSO:5% Cremophor-EL). Edema is reported as the change in paw diameter at 24 hr compared to the baseline. Thermal hyperalgesia was measured using the Hargreaves plantar apparatus (Ugo Basile) as previously reported [31].

### Formalin Test

Mice were habituated to the observation chamber (Plexiglas box, 25 cm×25 cm×25 cm) for 30 min prior to formalin injection. Mice were administered FABP inhibitors 60 min prior to formalin. The animals subsequently received an injection of formalin (2.5% in saline, 20 μl) into the plantar surface of the right hind paw using a 30 gauge needle and were immediately placed back into the observation chamber and nocifensive behavior (time spent licking or biting the paw) was recorded for 60 min. Nocifensive behavior was scored during the first phase (0–5 min) and the second phase (∼15–45 min) of the formalin test.

### Acetic acid-induced Writhing

Mice were acclimated to the laboratory environment for at least two hours and were habituated to the testing chamber for 30 min prior to acetic acid challenge. SBFI26, SBFI50, SBFI60, and SBFI62 (20 mg/kg) were administered via the subcutaneous route 60 min before acetic acid. Rimonabant, SR144528, GW6471, and naloxone were given 10 min before the inhibitors via the subcutaneous route. The mice subsequently received an i.p. injection of acetic acid (0.6% in sterile saline, 10 μl/g) and were placed in observation chambers. Abdominal stretches (marked by extension of the body and hind limbs) were counted for 20 min starting at 5 min post-injection.

### Locomotor Activity

Mice received injections of SBFI26 (20 mg/kg, i.p.) and 60 min later were placed in an observation chamber (46×25×20 cm) demarcated with 7×7 cm grids. Locomotor activity was recorded for 5 min by a video camera suspended above the chamber and subsequently scored by an observer blinded to the treatment conditions. A grid crossing was scored when both hind legs of the animal crossed the border of each grid square.

### Rectal temperature

Rectal temperature was measured at baseline and 90 min following vehicle or drug administration, by inserting a thermocouple probe 1.5 cm into the rectum and the temperature was read using a thermocouple meter (Kent Scientific).

### Rat neuropathic pain model

Male Fisher 344 rats were habituated and pre-injury evoked responses were obtained. The Hargreaves test was used to assess thermal hyperalgesia [36]. Briefly, plexiglass enclosures were set atop a plexiglass platform and infrared source set at 50% power located under the platform was used as the heat stimulus (Ugo Basile). A 200 mW, 535 nm diode laser was mounted in an adjustable stand placed beneath the plexiglass platform, and was used as an alternative heat stimulus for some of the animals in this study. The latency time to hind paw lift during heating of the plantar surface was recorded. Five recordings were obtained on each hind paw with at least 2 min rest between measurements. The maximum time for exposure to the infrared or diode laser source was 30.5 s to avoid any possible tissue injury. For mechanical threshold measurements, the animal enclosures rested on a screen with mesh of 0.5 cm spacing. Following acclimation, an electronic von Frey Anesthesiometer (IITC Life Sciences) was applied with increasing pressure to the plantar surface of the hind paw until the animals lifted the hind paw. The number of grams of force applied by the probe to induce withdrawal was recorded. Five recordings were obtained on each hind paw with at least 3 min between measurements. For CCI surgeries, our procedure closely followed that reported previously [37]. Briefly, each animal received an i.p. injection of ketamine/xylazine (75 mg/kg and 10 mg/kg, respectively). The hind leg was surgically prepped and the anesthetized animal placed prone on an sterile towel over a heating pad. The temperature was monitored rectally. A skin incision was made mid-thigh with a medium curved scalpel and then scissors were used to expose the sciatic nerve. Approximately 1 cm of the main trunk proximal to the trifuraction was isolated and 4 strands of 4-0 chromic gut were tied around the nerve ∼ 1 mm apart under magnification. In a modification of the original method, a 2-0 prolene strand was placed between the nerve and the gut to which the suture was tightened. The prolene strand was then removed. This prevented over tightening and reduced subsequent motor paralysis. The muscle layers were then reopposed and sutured, and surgical staples were used to close the skin. On post-injury days 7 through 10, heat and mechanical measurements were obained for the ipsilateral hindpaw of each CCI animal. Immediately following the post-injury measurements, animals were randomized and received i.p. injections of vehicle or 20 mg/kg SBI26 (DMSO:cremophor:sterile saline 1∶1∶2 by volume). Up to 0.8 ml of drug or vehicle was injected. The observer was blinded as to administration of drug or vehicle. Thermal and mechanical measurements were performed at 1 and 4 h post-injection. Data were normalized to pre-injury baseline measurements for each animal.

### Pharmacokinetics

Mice were injected with SBFI26 (20 mg/kg, i.p.) and were subsequently rapidly euthanized by decapitation at the indicated time points. The brain was rapidly removed and submerged in liquid nitrogen while the blood was collected into a K_2_-EDTA collection tube (Fisher). Plasma was isolated by centrifugation of the blood and 50 μl of the plasma was combined with 150 μl acetonitrile and centrifuged to remove the resulting protein pellet. Subsequently, 50 μl of water was added to 50 μl of the supernatant and 10 μl was injected for analysis into a Thermo TSQ Quantum Access Triple Quadrupole mass spectrometer (ThermoFisher). LC separation was achieved on a Luna C18 (150×2 mm) column. Mobile phase A consisted of 100% H_2_0 while mobile phase B was composed of 100% acetonitrile. The flow rate was 200 μl/min. A linear gradient was used and started at 50% B for 2 min, then ramped to 100% B in 8 min., with a 2 min hold at 100% B. The system was then equilibrated at 50% B for a cycle time of 20 min. The mass spectrometer was operated in the negative ion mode with the high voltage set at −3.5 kV. The sheath pressure was 20 and the capillary was set to 270 °C. Multiple reaction monitoring (MRM) was used with the transition *m/z* 421 to 277 at 29 eV as the quantitation channel, while *m/z* 421 to 205 at 17 eV was employed as a conformation channel. SBFI26 quantification was performed using an external calibration curve.

### Lipid Quantification

Quantification of endogenous NAEs and 2-AG were performed as previously described with minor modifications [30]. Mice were rapidly euthanized by decapitation and their brains flash frozen in liquid nitrogen. The brains were subsequently thawed, weighed, and homogenized in 8 ml of 2∶1∶1 CHCl_3_:MeOH:Tris (50 mM, pH 8) in the presence of 4 ng *d_4_*-PEA, 4 ng *d_2_*-OEA, 400 pg *d_4_*-AEA, and 40 ng *d_5_*-2-AG. Following centrifugation at 4°C, the organic layer was removed and brought up to 8 ml with the same buffer and centrifuged again. The resulting organic layer was dried down with argon and resuspended with 100 μl of 2∶1 CHCl_3_:MeOH and 10 μl was injected into the Thermo TSQ Quantum Access Triple Quadropole mass spectrometer. LC separation was achieved on a Gemini C18 (50×2 mm×5 μm) equipped with a Gemini C18 SecurityGuard precolumn (4 mm length ×2 mm internal diameter). Mobile phase A consisted of 95∶5% v:v H_2_0:MeOH while mobile phase B was composed of 60∶35∶5 v:v:v *i*-PrOH:MeOH:H_2_O and quantification was performed in the positive ion mode with the voltage set at 4 kV. 0.1% formic acid was added to assist in ionization. The sheath pressure was 30 and the capillary was set at 270 °C. The flow rate was 100 μl/min. The gradient started at 0% B and increased to 100% B over 15 min followed by an isocratic gradient of 100% B for 10 min, and was equilibrated for 15 min at 0% B.

### PPARα Transactivation Assay

PPARα activation was performed in HeLa cells as previously described [9].

### Statistical Analysis

Behavioral data are presented as means ± S.E.M. of at least 6 animals per group. Statistical significance between vehicle, inhibitor, and antagonist groups was determined using one-way ANOVA followed by Dunnett or Tukey post hoc analysis as appropriate. For CCI measurements, the data were analyzed using repeated measured ANOVA followed by Tukey post hoc analysis. In all cases, differences of p<0.05 were considered significant.

## Results

### Design and *in vitro* Evaluation of FABP Inhibitors

**Figure 1:**
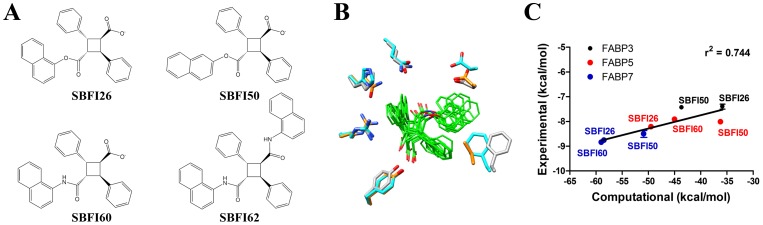
Structures and binding affinities of FABP inhibitors. (A) Chemical structures of FABP inhibitors. (B) Superimposed models of SBFI26, SBFI50, and SBFI60 in the binding pockets of FABP3, FABP5, and FABP7 reveal similar binding geometries. Amino acids lining the binding pockets are shown with teal, gold, and grey representing FABP3, FABP5, and FABP7, respectively. (C) Correlation between binding affinities and energy scores of SBFI26, SBFI50, and SBFI60 for FABP3, FABP5, and FABP7. Linear regression revealed an r^2^ value of 0.744. The binding affinities and energy scores are derived from Table 1 and Table 2.

The mammalian central nervous system expresses FABP3, FABP5, and FABP7 [38]. In a recent publication [31], we showed that pharmacological inhibition of FABPs with SBFI26, a compound with a high affinity for FABP5 and FABP7 reduced nociception in the formalin and carrageenan models of pain. To begin identifying the FABP subtypes mediating such antinociceptive effects, we designed three novel analogs of SBFI26 termed SBFI50, SBFI60, and SBFI62 (Fig. 1A), that display differential binding to FABP5 and FABP7. As an additional objective, the inhibitors were designed to exhibit limited binding to FABP3 because ablation of this protein has been previously shown to contribute to age-related cardiac hypertrophy [39].

SBFI50 was designed as a regioisomer of SBFI26 by coupling a 2-naphthol group with α-truxillic acid (Fig. 1A). In SBFI60, the ester bond between the α-truxillic acid and the naphthyl group of SBFI26 was replaced with an amide bond using naphthyl-1-amine. Computational analysis through energy-minimization suggested that although these three related inhibitors could be accommodated in the different FABP pockets as suggested by similar binding geometries (Fig. 1B), they would all possess higher affinities for FABP7 relative to FABP5 or FABP3 (Table 1). Encouragingly, these computational predictions showed a strong correlation (Figure 1C) with experimentally measured binding affinities (Table 2), suggesting the atomistic models employed in these studies can be used to explore different structure activity relationship series to optimize inhibitor potency.

**Table 1:** Single-point energy calculations of inhibitors docked to FABPs.

Compound	FABP3	FABP5	FABP7
	(kcal/mol)	(kcal/mol)	(kcal/mol)
SBFI26	−35.85	−49.45	−58.44
SBFI50	−43.64	−36.20	−50.88
SBFI60	−37.30	−44.97	−58.95

**Table 2:** Binding affinities of SBFI26, SBFI50, SBFI60, and SBFI62 to FABPs.

Compound	FABP3	FABP5	FABP7
	K_i_ (μM)	K_i_ (μM)	K_i_ (μM)
SBFI26	3.9±0.7	0.9±0.1	0.4±0.0
SBFI50	3.5±0.3	1.3±0.2	0.6±0.1
SBFI60	>10	1.6±0.0	0.3 ±0.0
SBFI62	2.6±1.1	3.3±0.7	6.1±0.5

The K_i_ values represent averages ± S.E. of three independent experiments.

Overall, compared to SBFI26, SBFI50 and SBFI60 possessed lower experimental affinities for FABP5, retained high affinities for FABP7, and were equally poor inhibitors of FABP3 (Table 2). Unexpectedly, SBFI60 proved to be an extremely weak inhibitor of FABP3 (K_i_>10 μM), suggesting that this compound may represent a novel scaffold for the design of future FABP5 and FABP7 selective inhibitors. An additional inhibitor, a dinaphthylamide analog of SBFI60 termed SBFI62 was also evaluated. Here, both of the carboxylic acids of α-truxillic acid were fully functionalized (Fig. 1A). Because SBFI62 lacks the free carboxylate previously determined to be important for interaction affinity [31], it was expected to exhibit low affinity across the FABP3, FABP5, and FABP7 series. A series of energy minimizations for this analog revealed that the added bulkier naphthylamide group would not allow for a conserved binding geometry shared by the other three inhibitors tested, which would similarly suggest lower affinity (data not shown). Indeed, when tested experimentally, SBFI62 showed much lower binding affinity for FABP5 and especially for FABP7 (Table 2).

### FABP Inhibitors Produce Antinociceptive Effects

**Figure 2:**
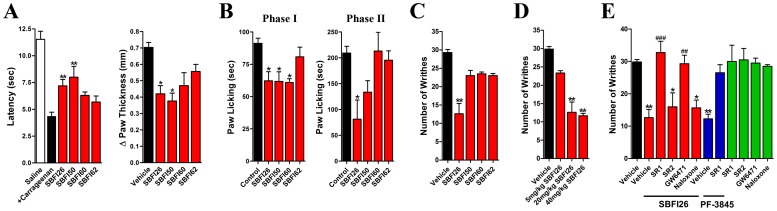
FABP inhibitors reduce nociception in models of inflammatory and visceral pain. (A) Effects of SBFI26, SBFI50, SBFI60, and SBFI62 (20 mg/kg, i.p.) upon carrageenan-induced thermal hyperalgesia (left panel) and paw edema (right panel) in mice. *, p<0.05; **, p<0.01 versus carrageenan injected animals (black bar) (n = 6). (B) Effect of FABP inhibitors (20 mg/kg, i.p.) upon the first (left panel) and second phases (right panel) of formalin-induced nociception in mice. *, p<0.05 versus vehicle control (n = 6). (C) SBFI26 reduces acetic acid-induced writhing in mice. **, p<0.01 (n = 6). (D) Dose-response of SBFI26-mediated inhibition of acetic acid writhing in mice. **, p<0.01 (n = 6). (E) The antinociceptive effects of SBFI26 are reversed by the cannabinoid receptor 1 antagonist rimonabant (SR1, 3 mg/kg) and the peroxisome proliferator-activated receptor alpha antagonist GW6471 (4 mg/kg). In contrast, the cannabinoid receptor 2 antagonist SR144518 (SR2, 3 mg/kg) and the opioid antagonist naloxone (2 mg/kg) were without effect. Rimonabant also reversed the antinociceptive effects of the FAAH inhibitor PF-3845 (blue bars). When administered alone, the antagonists did not modulate nociception (green bars). *, p<0.05; **, p<0.01 versus vehicle control. ##, p<0.01; ###, p<0.001 versus SBFI26 treated mice (n = 6–9).

The antinociceptive effects of the FABP inhibitors were examined using diverse models of nociception. In the carrageenan model of inflammatory pain, SBFI26 and SBFI50 (20 mg/kg, i.p.) reduced thermal hyperalgesia and paw edema while SBFI60 and SBFI62 were without effect (Fig. 2A). In the formalin model of inflammatory pain, SBFI26, SBFI50, and SBFI60 reduced the first phase of nociception while SBFI26 also reduced the second phase (Fig. 2B). Similar to the carrageenan model, SBFI62 was without effect. The antinociceptive effects of FABP inhibitors were also examined using the acetic acid writhing model of visceral pain. SBFI26 significantly reduced writhing induced by acetic acid while SBFI50, SBFI60, and SBFI62 were without effect (Fig. 2C). A dose response analysis revealed that SBFI26 exhibited near maximal efficacy at a dose of 20 mg/kg and increasing the dose did not significantly enhance the antinociceptive effects of this compound (Fig. 2D).

### CB_1_ and PPARα Receptors Mediate the Antinociceptive Effects of FABP Inhibitors

To examine the mechanism(s) mediating the analgesic effects of FABP inhibitors, mice were pretreated with the CB_1_ antagonist rimonabant, the CB_2_ antagonist SR144528, the PPARα antagonist GW6471, or the opioid receptor antagonist naloxone. Treatment of mice with rimonabant completely blocked the antinociceptive effects of SBFI26 in the acetic acid test (Fig. 2E), indicating that CB_1_ receptors mediate the effects of SBFI26. Rimonabant also blocked the antinociceptive effects of the FAAH inhibitor PF-3845, which is known to produce CB_1_-mediated analgesia by potentiating AEA signaling [40]. The antinociceptive effects of SBFI26 were also blocked by the PPARα antagonist GW6471 (Fig. 2E). In contrast, antagonism of CB_2_ receptors with SR144528 or opioid receptors with naloxone failed to reverse the antinociceptive effects of SBFI26. As expected, administration of the antagonists in the absence of SBFI26 did not alter nociceptive responses.

### FABP Inhibition Elevates Brain AEA Levels *in vivo*

**Figure 3:**
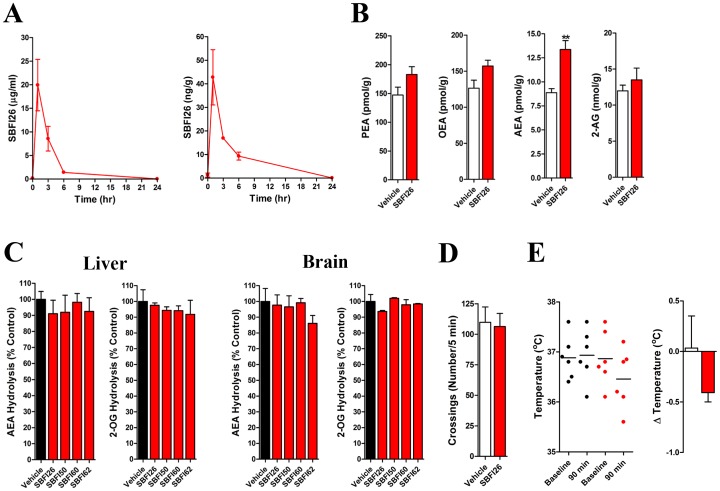
SBFI26 pharmacokinetics and its effect upon brain endocannabinoid levels. (A) Time course of SBFI26 levels in mouse plasma (left panel) and brain (right panel) following a single injection (20 mg/kg, i.p.) (n = 5). (B) SBFI26 (20 mg/kg, i.p.) elevates brain levels of the AEA. Tissues were harvested 90 min after SBFI26 administration. **, p<0.01 (n = 6). (C) Effect of FABP inhibitors (20 mg/kg, i.p.) upon AEA and 2-OG hydrolysis by mouse brain and liver homogenates following a single injection (n = 3). (D) SBFI26 (20 mg/kg, i.p.) does not alter locomotor activity in mice. Locomotor activity was measured 90 min after SBFI26 administration (n = 6). (E) SBFI26 (20 mg/kg, i.p.) does not reduce rectal temperature in mice. Left graph: scatterplot of rectal temperatures at baseline and 90 min following injection of vehicle (black circles) or SBFI26 (red circles). Right graph: change in rectal temperature between baseline and 90 min following administration of vehicle (white bar) or SBFI26 (red bar). Statistical analysis was performed by paired t-test (p = 0.205) (n = 6).

The data presented thus far suggest that FABP inhibitors produce analgesia by potentiating endocannabinoid/NAE signaling. To examine this directly, we first determined the plasma and brain levels of SBFI26. Following i.p. injection, plasma and brain levels of SBFI26 peaked within one hour and gradually decreased over time (Fig. 3A). The compound displayed a plasma and brain half-life of approximately 3 hrs with levels returning to baseline by 24 hrs. As expected, the brain levels were lower than the plasma levels.

The brain levels of PEA, OEA, AEA, and 2-AG were examined following SBFI26 administration. Injection of SBFI26 significantly elevated AEA levels while the levels of PEA, OEA, and 2-AG were unaffected (Fig. 3B). SBFI26 did not inhibit AEA hydrolysis (Fig. 3C), excluding the possibility that it elevates AEA levels by inhibiting FAAH. Similar results were found with the other FABP inhibitors. Furthermore, we have previously demonstrated that SBFI26 does not activate CB_1_ [31], indicating that its antinociceptive effects do not stem from direct activation of CB_1_. This is further supported by the inability of SBFI26 to reduce locomotor activity or rectal temperature (Figs. 3D and 3E), effects that are observed following administration of CB_1_ agonists [41], [42]. Collectively, these data are the first to demonstrate that inhibition of FABPs elevates brain AEA levels to produce analgesia.

### FABP Inhibitors Reduce Thermal Hyperalgesia Associated With Neuropathic Pain

**Figure 4:**
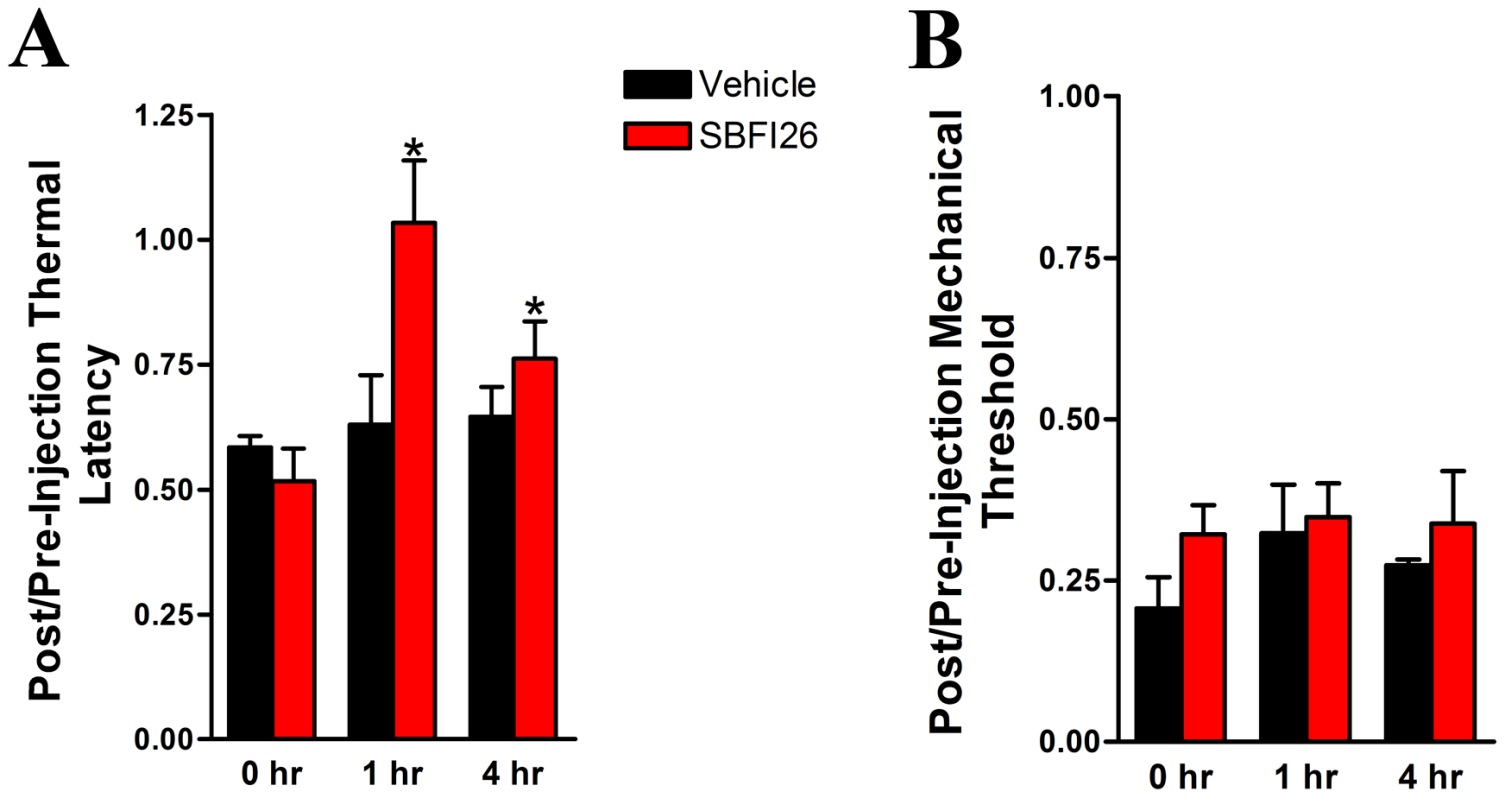
SBFI26 blocks thermal hyperalgesia in a rat model of neuropathic pain. (A) Thermal latencies and (B) mechanical thresholds in rats subjected to CCI were measured following injection of 20 mg/kg SBFI26. SBFI26 was injected at the 0 hr time point and evoked behaviors were measured at 1 and 4 hrs post-administration. The results are expressed as fraction of the pre-injury baseline measurement per animal (mean +/− SEM). Pre-injury thermal latency was 23.5 +/− 0.7 s and mechanical threshold was 29.9 +/− 0.6 g (mean +/− SEM). Results were analyzed by repeated measures ANOVA followed by Tukey's post-hoc analysis. *, p<0.05 versus 0 hr time point (n = 6).

To date, the antinociceptive effects of FABP inhibitors have been investigated in models of inflammatory pain. To ascertain whether FABP inhibitors reduce nociception associated with neuropathic pain, SBFI26 was administered to rats subjected to chronic constriction injury (CCI) of the sciatic nerve, a model of neuropathic pain. As expected, CCI rats developed thermal hyperalgesia and mechanical allodynia (Fig. 4A and B). Treatment with SBFI26 completely reversed thermal hyperalgesia at 1 hr post-administration with the effects persisting for up to 4 hrs (Fig. 4A). In contrast, SBFI50, which exhibits lower affinity for FABP5, was without effect (data not shown). Neither compound altered mechanical thresholds (Fig. 4B and data not shown).

## Discussion

Chronic pain affects approximately fifteen percent of the population [43], [44]. The endocannabinoid/NAE system has emerged as a promising target for the development of pain medications and cannabinoid receptor agonists have shown therapeutic efficacy in patients suffering from peripheral neuropathies [45]–[47]. However, cannabinoid receptor agonists suffer from untoward psychotropic effects [45], [48], highlighting the need to develop novel non-narcotic analgesics that lack the side-effects and abuse potential of cannabinoid receptor agonists and other clinically used analgesics.

Our group has recently identified FABPs as intracellular endocannabinoid/NAE binding proteins [8], [9]. Furthermore, we have demonstrated that pharmacological inhibition of FABPs produces antinociceptive effects that are mediated by cannabinoid receptors [31], suggesting that FABP inhibitors may reduce nociception by potentiating endocannabinoid signaling. To date, a direct demonstration that FABPs regulate endocannabinoid levels *in vivo* has not been reported. In addition, the development of FABP inhibitors as antinociceptive and anti-inflammatory agents requires demonstration of their broad efficacy in diverse models of pain. Here, we sought to begin addressing both of these outstanding questions.

In the current study, we show that inhibition of FABPs reduces nociception in models of inflammatory, visceral, and neuropathic pain. Of the inhibitors tested, SBFI26 was the most efficacious, followed by SBFI50 and SBFI60, with SBFI62 completely lacking efficacy. These results mirrored the experimental binding affinities of these compounds for FABP5 (Table 2), suggesting that high affinity binding to this protein may underlie the antinociceptive effects of FABP inhibitors. Indeed, SBFI50 and SBFI60, which exhibit lower affinity for FABP5 compared to SBFI26, were ineffective in the acetic acid test and similarly SBFI50 did not alter nociception in the CCI model. Furthermore, SBFI26, SBFI50, and SBFI60 bind to FABP7 with high affinity yet display markedly different *in vivo* efficacies, indicating that inhibition of FABP7 is unlikely to mediate the antinociceptive effects of these compounds. Future experiments employing mice lacking subsets of FABPs will be required to substantiate this hypothesis.

We have recently shown that AEA interacts with and is transported by FABPs to FAAH for catabolism [8], [9]. Indeed, knockdown of FABP5 in cultured cells reduced AEA uptake and hydrolysis [9]. Our current results are in agreement with these studies and constitute the first direct observation that FABP inhibition elevates brain AEA levels *in vivo*. Given that FABPs exhibit a more restricted expression profile compared to the global expression of FAAH in the brain [49], our data suggest that FABPs may be major regulators of the AEA tone in a subset of brain regions.

**Figure 5:**
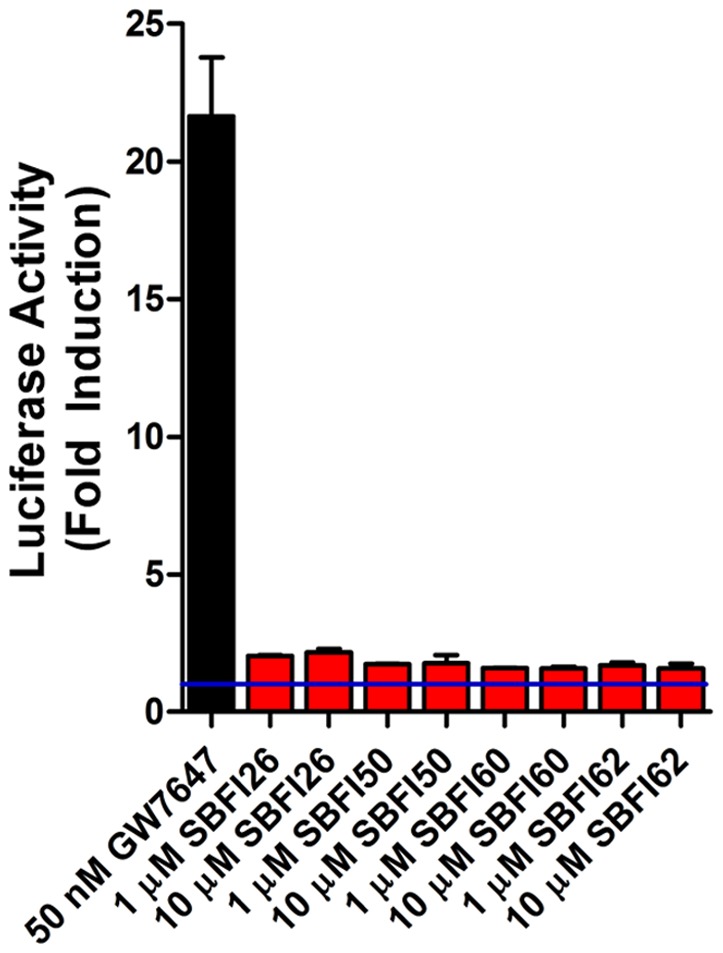
Activation of PPARα by FABP inhibitors. HeLa cells transfected with a PPARα reporter were incubated with the indicated compounds for 6 hours and luciferase and β-galactosidase activities were subsequently measured. Luciferase activity was normalized to β-galactosidase. The blue line represents baseline PPARα activation in the absence of agonists (n = 3).

NAEs activate diverse receptor systems, including cannabinoid and PPARα receptors [12], [13]. In addition to AEA, FABPs bind to other NAEs [9], suggesting that the antinociceptive effects observed in FABP inhibitor treated mice may be mediated by NAEs that engage cannabinoid and/or PPARα receptors. Indeed, our findings demonstrate the involvement of CB_1_ and PPARα receptors. It is noteworthy that inhibition of FABPs did not elevate PEA levels in mouse brains. Therefore, it is possible that the PPARα-mediated antinociceptive effects may stem from elevated PEA levels in peripheral nerves [29] or possibly in subsets of brain regions that regulate nociception, such that local changes in PEA levels may have eluded our analysis. Alternatively, FABPs may regulate the signaling of a lipid distinct from PEA that engages PPARα receptors. Lastly, it is unlikely that SBFI26 exerts its antinociceptive effects by directly activating PPARα. Indeed, while all four FABP inhibitors served as weak agonists at PPARα (Fig. 5), only SBFI26 exerted significant antinociceptive effects, which mirrors its higher affinity for FABP5.

Our findings also reveal that FABP inhibition modulates thermal hyperalgesia but not mechanical allodynia in the CCI model of neuropathic pain. Because heat and mechanical stimuli can be gated by non-overlapping peripheral sensory neurons [50]–[52], the selective modulation of heat sensitivity by FABPs may result from their expression in heat sensitive fibers and possibly their exclusion from mechanically-sensitive fibers. Alternatively, it is possible that greater CNS penetration of FABP inhibitors is required to modulate mechanical allodynia as reported for FAAH inhibitors [28].

**Figure 6:**
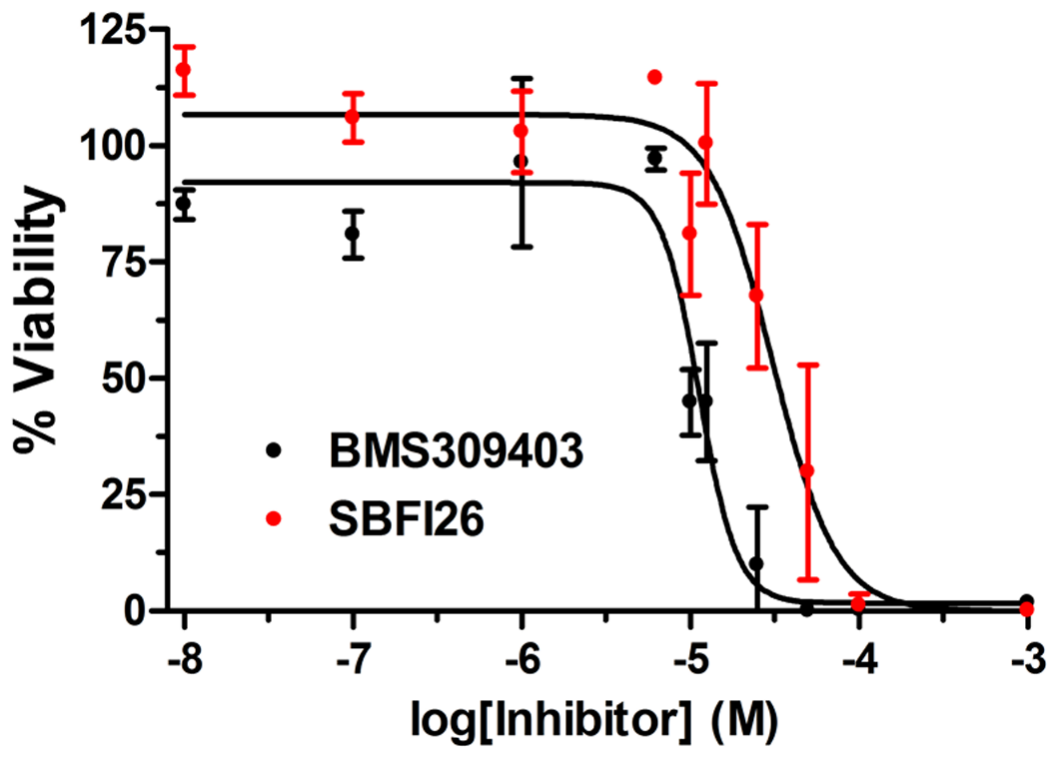
Cytotoxicity of SBFI26 and BMS309403 in HeLa cells. Cells were incubated with the indicated compounds and viability was assessed 24_50_ values of 31.1±4.1 μM and 11.3±0.9 μM, respectively (n = 3).

In conclusion, our findings demonstrate that FABP inhibitors suppress nociception in multiple pain models, effects that are mediated by both CB_1_ and PPARα receptors. Future medicinal chemistry efforts will be aimed at enhancing the potency, selectivity, and CNS penetration of FABP inhibitors. Because SBFI26 exhibits lower cytotoxicity compared to the previously described FABP inhibitor BMS309403 (Fig. 6) [53], inhibitors built upon this chemical scaffold may be better tolerated *in vivo*. Importantly, the compounds described herein weakly interact with FABP3, which may be advantageous as previous work has shown that mice lacking FABP3 develop age-dependent cardiac hypertrophy [39].
